# Image-Based Analysis Revealing the Molecular Mechanism of Peroxisome Dynamics in Plants

**DOI:** 10.3389/fcell.2022.883491

**Published:** 2022-05-03

**Authors:** Shino Goto-Yamada, Kazusato Oikawa, Katsuyuki T. Yamato, Masatake Kanai, Kazumi Hikino, Mikio Nishimura, Shoji Mano

**Affiliations:** ^1^ Małopolska Centre of Biotechnology, Jagiellonian University, Krakow, Poland; ^2^ Department of Material Chemistry, Graduate School of Engineering, Kyoto University, Kyoto, Japan; ^3^ Faculty of Biology-Oriented Science and Technology, Kindai University, Wakayama, Japan; ^4^ Department of Cell Biology, National Institute for Basic Biology, Okazaki, Japan; ^5^ Department of Biology, Faculty of Science and Engineering, Konan University, Kobe, Japan; ^6^ Department of Basic Biology, School of Life Science, SOKENDAI (The Graduate University for Advanced Studies), Okazaki, Japan

**Keywords:** *apem* mutant, *Arabidopsis thaliana*, imaging, *Marchantia polymorpha*, *peup* mutant, peroxisome

## Abstract

Peroxisomes are present in eukaryotic cells and have essential roles in various biological processes. Plant peroxisomes proliferate by *de novo* biosynthesis or division of pre-existing peroxisomes, degrade, or replace metabolic enzymes, in response to developmental stages, environmental changes, or external stimuli. Defects of peroxisome functions and biogenesis alter a variety of biological processes and cause aberrant plant growth. Traditionally, peroxisomal function-based screening has been employed to isolate *Arabidopsis thaliana* mutants that are defective in peroxisomal metabolism, such as lipid degradation and photorespiration. These analyses have revealed that the number, subcellular localization, and activity of peroxisomes are closely related to their efficient function, and the molecular mechanisms underlying peroxisome dynamics including organelle biogenesis, protein transport, and organelle interactions must be understood. Various approaches have been adopted to identify factors involved in peroxisome dynamics. With the development of imaging techniques and fluorescent proteins, peroxisome research has been accelerated. Image-based analyses provide intriguing results concerning the movement, morphology, and number of peroxisomes that were hard to obtain by other approaches. This review addresses image-based analysis of peroxisome dynamics in plants, especially *A. thaliana* and *Marchantia polymorpha*.

## 1 Introduction

Peroxisomes are present in eukaryotic cells and have important roles in various biological processes. In plants, peroxisomes are responsible for photorespiration, which is required to salvage byproducts of photosynthesis, and biosynthesis of plant hormones such as jasmonic acid and auxin, in addition to metabolism of fatty acids and detoxification of reactive oxygen species (ROS), which are common functions of peroxisomes in plant, mammalian, and yeast cells (Kamada et al., 2003). Peroxisomes are multiplied by division of pre-existing peroxisomes and degraded in response to developmental stages, environmental changes, and external stimuli. All peroxisomal proteins are encoded by the nuclear genome, and matrix proteins are transported to peroxisomes after translation in the cytosol. Many factors involved in the biosynthesis and functions of peroxisomes are conserved among various organisms. The factors responsible for biosynthesis of peroxisomes are collectively called PEROXINs (PEXs). More than 30 PEXs and their isoforms have been reported (Hu et al., 2012; Baker et al., 2016; Yuan et al., 2016; Fujiki et al., 2020; Jansen et al., 2021). However, some PEXs are unique to an organism. For example, the intraperoxisomal protein PEX8, PEX17, which is part of the docking complex on the peroxisomal membrane, and the PTS2 co-receptor PEX20 are reportedly involved in peroxisomal protein transport in fungi (Purdue et al., 1998; Agne et al., 2003; Montilla-Martinez et al., 2015; Jansen et al., 2021), but have not been identified in plants or animals. Fatty acid degradation via the β-oxidation pathway is a common type of metabolism in peroxisomes among various organisms. Although β-oxidation proceeds both in peroxisomes and mitochondria in mammalian cells, it occurs only in peroxisomes in plants and fungi (Poirier et al., 2006). Plant peroxisomes are also closely connected to photosynthesis, a unique plant system. The absolute byproduct glycolate-2-phosphate produced by RubisCO during photosynthesis is recycled to glycerate via photorespiration in peroxisomes and mitochondria to increase the photosynthetic efficiency (Peterhansel et al., 2010). In addition, peroxisomes are closely associated with chloroplasts when photosynthesis is active. Therefore, it is not sufficient to use information from yeast and animals to understand the molecular regulation that controls the morphology and dynamics of plant peroxisomes, and peroxisomal proteins in plants must be identified.

**FIGURE 1:**
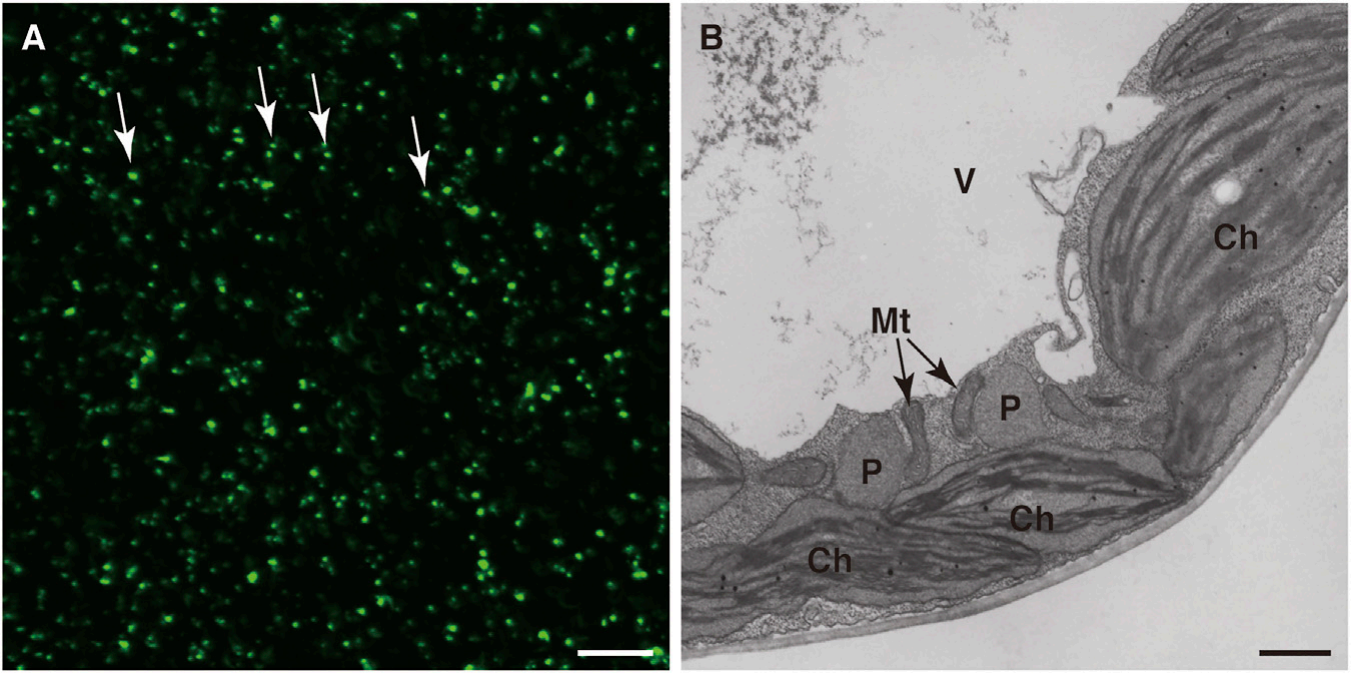
Detection of peroxisomes in leaf cells. Fluorescence microscopic analysis of GFP **(A)** and electron microscopic analysis **(B)** were performed of transgenic *A. thaliana* (GFP-PTS1) expressing the fusion gene of *GFP* with *PTS1* under the regulation of the constitutive promoter. **(A)** A lot of peroxisomes were visualized as spherical structures (Jedd and Chua, 2002; Mano et al., 2002; Mathur et al., 2002). Some representative peroxisomes are indicated by arrows. Bar, 20 µm. **(B)** Transmission electron microscopic observation of GFP-PTS1 plants (Mano et al., 2002). P, peroxisome; Mt, mitochondrion; Ch, chloroplast; V, vacuole. Bar, 1 µm.

Peroxisome research has been accelerated by the application of imaging techniques such as the use of fluorescent proteins. In 2002, three groups visualized peroxisomes with GFP (Figure 1A; Jedd and Chua, 2002; Mano et al., 2002; Mathur et al., 2002). Visualization of peroxisomes was simple and did not affect their functions or dynamics. It only required expression of the fusion gene encoding peroxisome targeting signal (PTS) 1 or PTS2 added to the C- or N-terminus of GFP, respectively. Additional reagents and treatments were not required to observe GFP-labeled peroxisomes. Observation of GFP-labeled peroxisomes under a fluorescence microscope provided important information about peroxisome dynamics such as their morphology, number, size, intracellular distribution, movement, and interactions with other subcellular components, which was hard to obtain by traditional approaches. In particular, live imaging is a powerful technique in the plant peroxisome research field and provides useful information such as the velocity, direction of movement, and morphological changes of peroxisomes (Supplementary Movie S1; Jedd and Chua, 2002; Mano et al., 2002; Mathur et al., 2002). In those days, electron microscopic analysis was the only way to observe peroxisome dynamics, especially their shape and size. This is because, unlike mitochondria and other organelles, there are no dyes to specifically stain peroxisomes and, unlike chloroplasts, peroxisomes do not emit autofluorescence, which is occasionally used to monitor chloroplast dynamics in living cells. Electron microscopic analysis has been a powerful tool to investigate ultrafine structures of peroxisomes (Figure 1B). However, electron micrographs are static images and therefore do not provide temporal information. Meanwhile, although the resolution of fluorescence images is inferior to that of electron micrographs, researchers can obtain spatiotemporal information from observations under a fluorescence microscope. A confocal laser scanning microscope can generate 3D images containing information about the distribution of peroxisomes in the whole cell.

Thus, it has become easier for researchers to obtain information about the dynamics of peroxisomes in plant cells using imaging analysis. Peroxisomes are maintained by sophisticated machinery that regulates their biogenesis and functions, such as their elongation, division, and protein transport. Disturbance of these regulatory mechanisms can cause peroxisome anomalies in cells. This is expected to result in abnormal peroxisomes, and the introduction of fluorescent peroxisome markers allows direct observation of such aberrations. For example, if the mechanisms controlling peroxisome proliferation, such as their elongation and division, were defective, peroxisomes with an abnormal size and morphology would be observed. If the efficiency of peroxisomal protein transport was decreased, GFP fluorescence would be observed in the cytosol as well as in peroxisomes. Based on these insights, *Arabidopsis thaliana aberrant peroxisome morphology* (*apem*) mutants were isolated and analyzed, which provided useful information about peroxisome dynamics (Mano et al., 2004; Mano et al., 2006; Goto et al., 2011; Goto-Yamada et al., 2014a). These studies are described in Section 2.2 in more detail. Among various important peroxisome functions, photorespiration is a metabolic system involving reactions in chloroplasts, mitochondria, and peroxisomes, and interactions among these three organelles support efficient photorespiratory activity (Oikawa et al., 2015). *A. thaliana peroxisome unusual positioning* (*peup*) mutants were screened based on an abnormal pattern of peroxisome positioning in cells (Shibata et al., 2013). In this screening, green and red fluorescence, which was derived from GFP-labeled peroxisomes and autofluorescence emitted by chloroplasts, was used to analyze the intracellular distributions of both organelles. The detailed studies of *peup* mutants are described in Section 2.3.

As described in Section 3, the liverwort *Marchantia polymorpha* has become a useful model plant for plant life science research due to several advantages, such as the availability of well-developed genetic resources and methods to introduce exogeneous genes for visualizing subcellular compartments and to perform genome editing with the CRISPR/Cas9 system (Bowman, 2016; Bowman et al., 2017; Iwasaki et al., 2021; Kohchi et al., 2021). Like in *A. thaliana*, peroxisomes are easily visualized with fluorescent proteins in *M. polymorpha*, and therefore *M. polymorpha* is becoming a useful material in the plant peroxisome research field (Ogasawara et al., 2013; Kimura and Kodama, 2016; Mano et al., 2018). By comparing the molecular mechanisms regulating peroxisome dynamics in *A. thaliana* and *M. polymorpha*, we can determine whether mechanisms related to plant peroxisomes are conserved among all plant species or are specific to particular plant species.

This review comprehensively addresses image-based analysis of peroxisomes. In particular, we describe the identification and characterization of factors involved in peroxisome dynamics based on analyses of mutants with peroxisome defects in *A. thaliana*, and a combination of imaging and bioinformatics analyses in *M. polymorpha*.

## 2 Imaging Analysis of *A. thaliana* Mutants With Peroxisome Defects

### 2.1 Introduction of Image-Based Screening to Identify New Mutants With Peroxisome Defects

The forward genetics approach to identify novel mutants that display an abnormality of peroxisomes followed by characterization of the gene products responsible is a powerful method to improve our knowledge of peroxisome dynamics, metabolism, and biosynthesis. Efficient isolation to obtain promising mutants is key for successful research. The model flowering plant *A. thaliana* has been used to screen mutants with peroxisome defects because genetic resources and information are abundant (Koornneef and Meinke, 2010). Various approaches have been adopted to identify mutants with peroxisome defects. Traditionally, peroxisomal function-based screening has been performed to identify a number of mutants that are defective in lipid metabolism and photorespiration, contributing to the identification of several peroxisome-related genes, such as those encoding enzymes involved in metabolism and *PEX*s (Somerville and Ogren, 1980; Somerville and Ogren, 1981; Hayashi et al., 1998; Hayashi et al., 2000; Zolman et al., 2000; Zolman et al., 2001; Hayashi et al., 2002; Zolman and Bartel, 2004). Screening relied on morphological differences from wild-type (WT) plants, such as dwarfism and short roots, as a result of indirect effects. To obtain novel mutants with peroxisome defects, including peroxisome dynamics-deficient mutants, another screening approach is employed: visualized peroxisome-based mutant screening. The first set of mutants, called the *apem* mutant series, was isolated by focusing on plant peroxisome dynamics, i.e., their morphology, movement, number, and subcellular localization (Table 1). As a supplementary note, the abbreviation *apm* was initially used, but has been replaced with *apem* to avoid confusion with other *A. thaliana* mutants. The mutants were screened from the pool of ethyl methanesulfonate (EMS)-mutagenized *A. thaliana* (accession Columbia) plants, which expressed the peroxisome marker *GFP-PTS1*, based on a GFP fluorescence pattern that differed from that in WT plants (Mano et al., 2002). Approximately 37,000 M2 plants were examined under a fluorescence microscope, and 82 mutants were isolated. These mutants were classified into four groups: 1) elongated peroxisomes, 2) enlarged peroxisomes, 3) mislocalization of GFP-PTS1 protein to the cytosol, and 4) other distributions of GFP (Mano et al., 2004; Mano et al., 2006; Goto et al., 2011; Goto-Yamada et al., 2014a). In addition, the same mutagenized seed pool was screened for differences in the pattern of interactions between peroxisomes and chloroplasts. In these mutants, designated *peup*, the size and morphology of peroxisomes were almost identical to those in the parent plants, but the intracellular distributions of peroxisomes and chloroplasts were dramatically altered (Shibata et al., 2013; Goto-Yamada et al., 2019). Apart from *apem* and *peup* mutants, screenings based on visualized peroxisomes were also reported by other groups (Zhang and Hu, 2009; Rinaldi et al., 2016). In addition, Lingard et al. (2009) used *GFP* fused with *ISOCITRATE LYASE* (*ICL*), which encodes a glyoxylate cycle enzyme in peroxisomes, under the regulation of the *ICL* promoter to investigate peroxisome-associated protein degradation (Lingard et al., 2009; Burkhart et al., 2013). In this section, we introduce imaging analysis-based peroxisome research. We first outline various *apem* and *peup* mutants, and then describe reports in *A. thaliana* in comparison with other organisms.

**TABLE 1:** Phenotypes and causative genes in *apem* and *peup* mutants.

Mutant name	Peroxisome phenotype	AGI code	Gene name	Mutation	Reference
*apem1*	Elongated peroxisomes	At4g33650	DYNAMIN-RELATED PROTEIN 3A	D172N (*apem1-13*) and 11 other alleles	Mano et al. (2004)
*apem2*	Accumulation of peroxisomal proteins in the cytosol	At3g07560	PEROXIN 13	Q263stop	Mano et al. (2006)
*apem3*	Enlarged peroxisomes	At2g39970	PEROXISOMAL MEMBRANE PROTEIN 38, PEROXISOMAL NAD CARRIER	W60stop	Mano et al. (2011)
*apem4*	Accumulation of peroxisomal proteins in the cytosol	At3g04460	PEROXIN 12	R170K	Mano et al. (2006)
*apem9*	Accumulation of peroxisomal proteins in the cytosol	At3g10572	ABERRANT PEROXISOME MORPHOLOGY 9, PEROXIN 26, PEROXIN 15	G278E	Goto et al. (2011)
*apem10*	Accumulation of peroxisomal proteins in the cytosol, decreased number of peroxisomes, and enlarged peroxisomes	At5g47040	LON PROTEASE 2	Q144stop	Goto-Yamada et al. (2014)
*peup1*	Increased number of peroxisomes	At3g19190	AUTOPHAGY-RELATED PROTEIN 2	W1309stop (*peup1-1*) and another allele	Shibata et al. (2013)
*peup2*	Increased number of peroxisomes	At3g62770	AUTOPHAGY-RELATED PROTEIN 18A	Q384stop	Shibata et al. (2013)
*peup4*	Increased number of peroxisomes	At5g45900	AUTOPHAGY-RELATED PROTEIN 7	C536Y	Shibata et al. (2013)
*peup17*	Increased number of peroxisomes	At5g17290	AUTOPHAGY-RELATED PROTEIN 5	Splice donor site between the third exon and third intron	Goto-Yamada et al. (2019)
*peup22*	Increased number of peroxisomes	At5g45900	AUTOPHAGY-RELATED PROTEIN 7	Q522stop	Goto-Yamada et al. (2019)

All mutants in this list were obtained from the pool of ethyl methanesulfonate (EMS)-mutagenized A. thaliana (accession Columbia) plants, which expressed the peroxisome marker GFP-PTS1.

### 2.2 Analysis of Peroxisome Biogenesis, Proliferation, and Quality Control With *apem* Mutants

#### 2.2.1 *apem1*/*drp3a*

**FIGURE 2:**
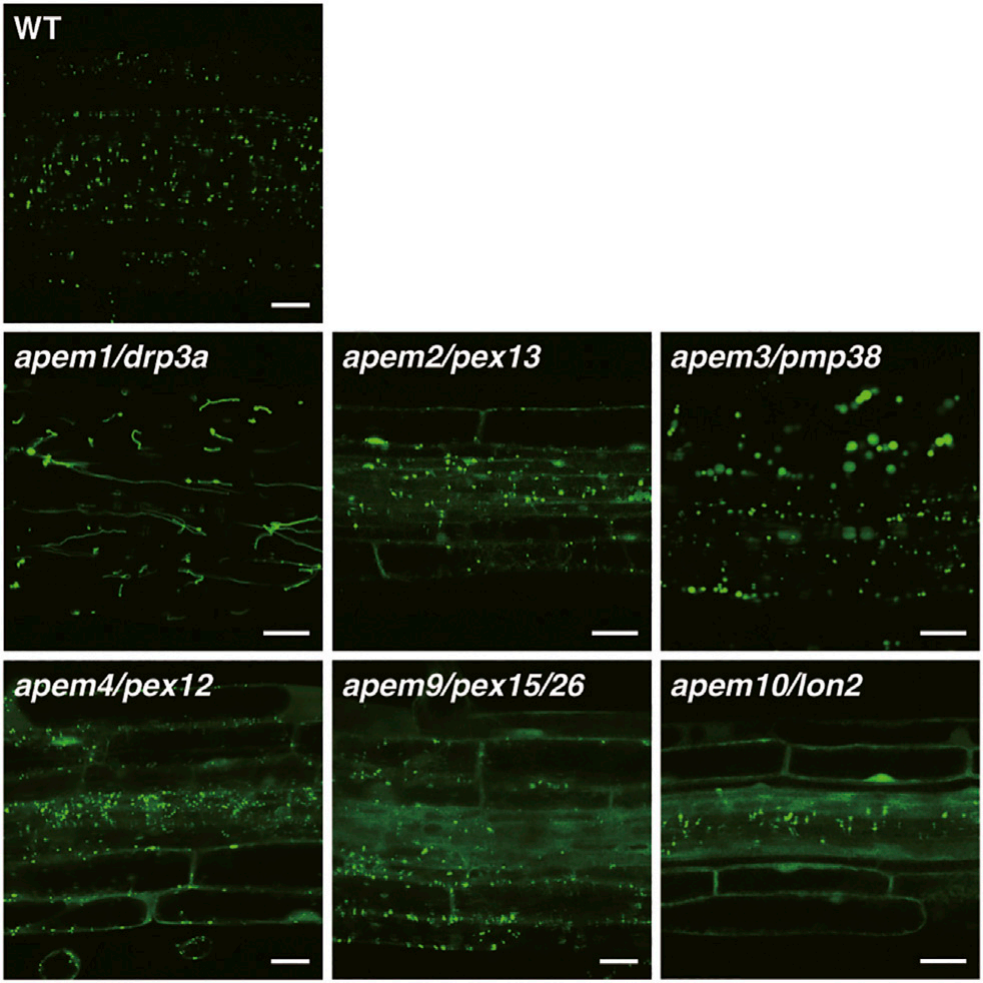
GFP fluorescence in root tissue of the WT plant and *apem* mutants expressing the peroxisome marker *GFP-PTS1* (Mano et al., 2004; Mano et al., 2006; Goto et al., 2011; Goto-Yamada et al., 2014a). Bars, 20 µm.

The *apem1* (previously known as *apm1*) mutant exhibits elongated and a reduced number of peroxisomes in a variety of cells throughout the plant (Figure 2). Mitochondria are also elongated, but other organelles such as chloroplasts, nuclei, the Golgi apparatus, and the endoplasmic reticulum (ER) are not. The *APEM1* gene encodes DYNAMIN-RELATED PROTEIN 3A (DRP3A), a member of the dynamin superfamily that has a pivotal role in vesicle division and organelle fission and fusion (Mano et al., 2004; Praefcke and McMahon, 2004). In addition to DRP3A, its closest homolog, DRP3B, is also involved in peroxisome and mitochondria fission, and plant- and alga-specific DRP5B affects peroxisome, chloroplast, and mitochondria fission (Fujimoto et al., 2009; Aung and Hu, 2012). Interestingly, forward genetic screening isolated a number of independent lines possessing mutations at the *DRP3A* locus (Mano et al., 2004; Praefcke and McMahon, 2004), but not the *DRP3B* or *DRP5B* locus (Aung and Hu, 2012). Various experimental data indicate that DRP3A is the primary protein responsible for peroxisome fission (Fujimoto et al., 2009; Zhang and Hu, 2009; Aung and Hu, 2012).

#### 2.2.2 *apem2*/*pex13*

In the *apem2* (previously known as *apm2*) mutant, GFP-PTS1 protein is located in the cytosol as well as in peroxisomes (Figure 2). The *APEM2* gene encodes the integral peroxisomal membrane protein PEX13 (Mano et al., 2006). Peroxisomal matrix proteins are transported to peroxisomes by their receptor PEX5 or PEX7, and translocate into the peroxisomal matrix through the pore formed by the receptor and the docking complex consisting of PEX14 and PEX13 (and PEX17 in fungi) on the peroxisomal membrane. The *apem2* mutation introduces a stop codon at position 263 instead of glutamine in the C-terminal region. The C-terminus of PEX13 interacts with PEX14 and PEX5 via the Src homology 3 (SH3) domain in fungi (Bottger et al., 2000; Douangamath et al., 2002). However, *A. thaliana* PEX13 lacks an obvious SH3 domain, and a yeast two-hybrid assay showed that PEX13 interacts with PEX7, but not PEX14 or PEX5 (Mano et al., 2006; Boisson-Dernier et al., 2008). Another group reported a different *pex13* mutation in which Glu is substituted by Lys only 20 amino acids upstream of the *apem2* mutation (Woodward et al., 2014), supporting the importance of the C-terminal region of plant PEX13. Boisson-Dernier et al. (2008) isolated the *A. thaliana abstinence by mutual consent* (*amc*) mutant, which disrupts male-female gametophyte recognition (Boisson-Dernier et al., 2008). *amc* is a PEX13 loss-of-function mutant, showing that peroxisomes play a role in the reproductive process (Boisson-Dernier et al., 2008; Goto-Yamada et al., 2014b). These results indicate that PEX13 and other peroxisomal biogenesis factors evolved differently in plants in comparison with other organisms.

#### 2.2.3 *apem3*/*pmp38*/*pxn*

The *apem3* mutant exhibits enlarged peroxisomes, and the diameter of some can reach more than 10 µm (Figure 2; Mano et al., 2011). Introduction of the *apem1/drp3a* mutation, which impairs peroxisome division, into *apem3* does not affect the enlarged peroxisome phenotype, and DRP3A protein is detected on *apem3* enlarged peroxisomes. In addition, division-arrested *apem1*/*drp3a* peroxisomes do not show the same level of enlargement as those in the *apem3* mutant (Mano et al., 2011). These results suggest that the enlarged peroxisomes observed in *apem3* do not arise due to perturbation of peroxisome division. The *APEM3* gene encodes PEROXISOMAL MEMBRANE PROTEIN 38 (PMP38), which is also known as PEROXISOMAL NAD^+^ CARRIER (PXN) because it can transport NAD^+^ into the peroxisomal matrix (Fukao et al., 2001; Eubel et al., 2008; Bernhardt et al., 2012). Blockade of NAD^+^ supply reduces the efficiency of lipid metabolism in peroxisomes and induces the accumulation of long-chain fatty acids (Bernhardt et al., 2012). Other mutants defective in fatty acid β-oxidation also contain enlarged peroxisomes (Hayashi et al., 2001). In addition, the enlargement of peroxisomes in the *pmp38*/*pxn* mutant is suppressed by disruption of PEROXISOMAL ABC TRANSPORTER1 (PXA1), which transports fatty acids into peroxisomes (Rinaldi et al., 2016). These results demonstrate that the enlargement of peroxisomes in *apem3*/*pmp38*/*pxn* mutants is due to the accumulation of fatty acids, which may produce hydrogen peroxide and damage peroxisomes (Rinaldi et al., 2016).

#### 2.2.4 *apem4*/*pex12*

In the *apem4* (previously known as *apm4*) mutant, GFP-PTS1 protein is located in the cytosol as well as in peroxisomes (Figure 2). The *APEM4* gene encodes PEX12 (Mano et al., 2006), which is one of the RING-finger domain-containing peroxins (PEX2, PEX10, and PEX12) involved in peroxisomal protein transport. In fungi, mono-ubiquitination of the peroxisomal protein receptor Pex5 is mediated by Pex4 and Pex12, which are E2 and E3 ligases, respectively (Platta et al., 2009), and this is required for recycling of Pex5 from the peroxisomal membrane to the cytosol. Three *A. thaliana* RING peroxins, PEX2, PEX10, and PEX12, exhibit E3 ubiquitin ligase activity *in vitro* (Kaur et al., 2013). Indeed, the *apem4/pex12* mutant displayed accumulation of PEX5 protein in the peroxisomal membrane fraction, while the *pex12-1* mutant exhibited elevated PEX5 and PEX7 levels (Mano et al., 2006; Kao et al., 2016). The *apem4* mutant, as well as another *pex12* mutant, exhibits suppression of not only PTS1- but also PTS2-directed protein transport. These results indicate that a PEX12 defect impairs PEX5 and PEX7 recycling. *A. thaliana* PEX12 can bind to PEX7 (Singh et al., 2009). Therefore, a defect of PEX12 also can lead to a decrease in the efficiency of PEX7-dependent PTS2 transport. Interestingly, the *apem4* mutation, which replaces Arg170 with Lys, is adjacent to the *pex12-1* mutation, which replaces Glu171 with Lys (Mano et al., 2006; Kao et al., 2016). However, these amino acid residues are not conserved among organisms, and the function of the region containing these two amino acid residues has not been clarified. The findings that mutations at two positions affect peroxisome transport imply the specific function of this region of PEX12.

#### 2.2.5 *apem9*/*pex15*/*pex26*

Like *apem2* and *apem4*, the *apem9* mutant was isolated on the basis of a phenotype in which peroxisomal proteins accumulate in the cytosol and exhibits defects in both PTS1- and PTS2-dependent transport (Figure 2; Goto et al., 2011). *APEM9* encodes a protein of unknown function, homologs of which are found only in plant genomes. Hydropathy profile analysis of APEM9 suggested that it is similar to yeast PEX15, which can recruit the PEX1/PEX6 complex from the cytosol to peroxisomal membranes (Goto et al., 2011). PEX15 is a tail-anchored peroxisomal membrane protein that is involved in recycling of PEX5 in fungi (Birschmann et al., 2003), and PEX26 was reported to be a PEX15 ortholog in mammalian cells (Matsumoto et al., 2003). The secondary structure of APEM9 appears to be more similar to that of PEX26 than PEX15, although sequence similarity is quite low (Goto et al., 2011). The AAA+ ATPases PEX1 and PEX6 form a heterooligomer and can function as an unfoldase to extract PEX5 from the membrane (Ciniawsky et al., 2015; Gardner et al., 2018; Pedrosa et al., 2018). The *apem9* mutation substitutes Gly278 with Glu in the transmembrane domain, which affects the peroxisomal localization of APEM9 and the PEX1/PEX6 complex (Goto et al., 2011). DAYU (a synonym of APEM9) binds to PEX13 and PEX16 (Li et al., 2014). As described above, PEX13 is a component of the PEX5 docking complex, and bridging the docking complex closer to the recycling machinery may make export of PEX5 efficient. Unlike *apem2*/*pex13*, mutants defective in APEM9/PEX15/PEX26 or PEX6 displayed a decreased amount of PEX5, and the PEX5 level was increased by treatment with the proteasome inhibitor MG132, suggesting that PEX5 undergoes proteasomal degradation when recycling machinery does not extract it properly (Gonzalez et al., 2017).

#### 2.2.6 *apem10*/*lon2*

The *apem10* mutant exhibits a decreased level of punctate peroxisomal GFP signals and accumulation of GFP fluorescence in the cytosol (Figure 2). The *apem10* mutation replaces Gln144 with a stop codon in the peroxisomal LON PROTEASE 2 (LON2) protein. Immunostaining of the peroxisomal membrane protein ASCORBATE PEROXIDASE (APX) showed that the number of peroxisomes is reduced in the *apem10* mutant (Goto-Yamada et al., 2014a). This indicates that peroxisomes are degraded and that matrix proteins, such as GFP-PTS1, accumulate in the cytosol (Lingard and Bartel, 2009; Goto-Yamada et al., 2014a). The *apem10/lon2* phenotype is accentuated with age. Accumulation of GFP-PTS1 in the cytosol is not observed in newly emerging young leaves, but is prominent in well-developed mature leaves in which peroxisomes are enlarged occasionally (Farmer et al., 2013; Goto-Yamada et al., 2014a). In addition, the phenotype of enlarged peroxisomes in *apem10* mutants was suppressed under high CO_2_ conditions, in which the photorespiratory pathway was not required, indicating that the *apem10* phenotype correlates with peroxisomal activity (Goto-Yamada et al., 2014a). Induction of autophagy deficiency rescued the *apem10/lon2* phenotype (Farmer et al., 2013; Goto-Yamada et al., 2014a). Peroxisomal metabolic systems contain a variety of oxidases and produce hydrogen peroxide, which is a threat to peroxisomal proteins and membranes (Nishimura et al., 1983; Corpas et al., 2020). Studies of plant LON2 and autophagy revealed the existence of two independent peroxisome maintenance processes: 1) LON2 degrades abnormal and/or obsolete matrix proteins inside peroxisomes and 2) when peroxisomes are not sufficiently restored by LON2, autophagy degrades abnormal peroxisomes (Farmer et al., 2013; Shibata et al., 2013; Goto-Yamada et al., 2014a). A lack of LON2 induces peroxisome degradation via autophagy (called pexophagy). Plant peroxisomes alter their metabolic systems in response to their environment and developmental changes. The molecular mechanisms to replace enzymes responsible for each type of metabolism have long been discussed. The quality control system of peroxisomes, which involves the two aforementioned coordinated degradation processes, explains the mechanism underlying peroxisomal functional transition and a new model was proposed (Goto-Yamada et al., 2015). Interestingly, the protease activity of the C-terminal serine peptidase domain seems to contribute to degradation of peroxisomal proteins, but not to inhibition of pexophagy, which is dependent on the N-terminal chaperone domain (Goto-Yamada et al., 2014a). The mechanisms underlying inhibition and induction of pexophagy remain to be investigated.

### 2.3 Analysis of Organelle-Organelle Interactions

#### 2.3.1 Physical Interactions of Peroxisomes With Other Organelles

Leaf peroxisomes function in many metabolic pathways, some of which also involve other organelles such as mitochondria and chloroplasts (Mano and Nishimura, 2005; Hayashi and Nishimura, 2006; Nyathi and Baker, 2006; Hu et al., 2012; Kao et al., 2018; Oikawa et al., 2019). Therefore, it is thought that the close localization of peroxisomes, mitochondria, and chloroplasts contributes to efficient metabolite flow. In fact, electron micrographs showed these three organelles in close contact with each other (Frederick and Newcomb, 1969; Tolbert, 1982; Nishimura et al., 1986; Oikawa et al., 2019; Baillie et al., 2020). As described above, visualization of peroxisomes using fluorescent proteins enables analysis of their positioning in living cells (Jedd and Chua, 2002; Mano et al., 2002; Mathur et al., 2002). Peroxisomes actively move on actin filaments using myosin motors and interact with other organelles such as chloroplasts and mitochondria (Jedd and Chua, 2002; Mano et al., 2002; Mathur et al., 2002; Goto-Yamada et al., 2015; Oikawa et al., 2015; Oikawa et al., 2019; Baillie et al., 2020; Mathur, 2021). Peroxisomes in dark-adapted cells change their shape from spherical to elliptical in order to strengthen their interactions with chloroplasts under the photosynthetic condition (Oikawa et al., 2015; Oikawa et al., 2019). The strength of interactions between peroxisomes and chloroplasts in the dark and light was measured using a femtosecond laser to evaluate adhesion strength directly in living leaf cells. This revealed that light has a strong positive effect on adhesion (Oikawa et al., 2015; Hosokawa et al., 2016). An optical tweezer was used to measure the interaction strength between a peroxisome and a chloroplast *in vitro* (Gao et al., 2016). These studies revealed the existence of a physical interaction between peroxisomes and chloroplasts, and suggest that this interaction has physiological significance for plant cellular function.

The tethering factor(s) that connects a peroxisome and a chloroplast remains unclear, but PEX10, a C3HC4 zing RING-finger peroxisomal membrane protein, is one candidate (Schumann et al., 2007). Expression of dominant-negative PEX10 disturbed the interaction of peroxisomes with chloroplasts and photorespiration. Further studies are required to clarify whether PEX10 functions as a tethering factor between a peroxisome and a chloroplast directly and whether other PEXs are involved in this interaction.

It was recently reported that a large complex of glycolysis enzymes, a phosphoglycerate mutase-enolase metabolon, plays a role in the interaction between mitochondria and chloroplasts (Zhang et al., 2020). A direct interaction between mitochondria and chloroplasts has been clearly shown by analyzing mitochondrial movement (Oikawa et al., 2021). It is interesting to investigate whether enzymes in the metabolite pathway participate in the interaction between peroxisomes and chloroplasts similar to the interaction between mitochondria and chloroplasts. Determination of the mechanism underlying the peroxisome-chloroplast interaction will help to elucidate the role of organelle interactions in plants.

Glyoxysomes, one of the peroxisomes, engage in the degradation of reserve oil stored in the oil body via β-oxidation and the glyoxylate cycle. *A. thaliana peroxisome defective 1* (*ped1*) was defective in fatty acid β-oxidation (Hayashi et al., 1998). Detailed electron microscopic analysis revealed that the glyoxysomes in etiolated cotyledons of the *ped1* mutant appeared abnormal, having tubular structures that are derived from invagination of the glyoxysomal membrane. (Hayashi et al., 2001). These invagination sites were always in contact with oil bodies, proposing that direct interaction between glyoxysomes and lipid bodies is involved in the process of fatty acid metabolism (Hayashi et al., 2001). *A. thaliana sugar dependent 1* (*sdp1*) mutant was identified from the pool of ethyl methanesulfonate (EMS)-mutagenized *A. thaliana*, which expressed the fusion gene encoding OLEOSIN, one of oil body membrane proteins, with GFP, as having larger and more oil body aggregates compared with the wild-type plant (Cui et al., 2016). SDP1 is a triacylglycerol (TAG) lipase that resides on the oil body membrane, and hydrolyzes TAG to produce fatty acids. From the analyses using the *sdp1* mutant, Cui et al. (2016) showed that sucrose is a key factor for peroxisome-oil body interaction dependent on actin filaments, and that PEROXISOME DEFFECTIVE 3 (PED3), a peroxisomal ATP binding cassette transporter, is the potential anchor protein to the membranes of these organelles (Cui et al., 2016).

The analysis of mutants accumulating excess peroxisomes described below and several other reports indicate that autophagic peroxisomal degradation, or pexophagy, is one of the major peroxisomal quality control mechanisms, along with maintenance by the chaperone-proteinase LON2/APEM10 (Farmer et al., 2013; Kim et al., 2013; Shibata et al., 2013; Goto-Yamada et al., 2014a). Mutants with defective autophagy fail to form autophagosomes and subsequently degrade peroxisomes. In these mutants, the cisterna-like membrane structure associated with peroxisomes and the ATG8 protein, one of the autophagosome components, were detected on autophagosome membrane structures by immunoelectron microscopy (Yoshimoto et al., 2014). Reduction-oxidation sensitive green fluorescent protein (roGFP) analysis revealed that the peroxisomes of autophagy-deficient mutants are highly oxidized, and that mCherry-ATG8a proteins selectively assemble on the oxidized peroxisomes (Shibata et al., 2013). Peroxisomes are oxidized by hydrogen peroxide produced in the process of peroxisome function, and such damaged peroxisomes are selectively recognized and eliminated by autophagy.

#### 2.3.2 *peup* Mutants

**FIGURE 3:**
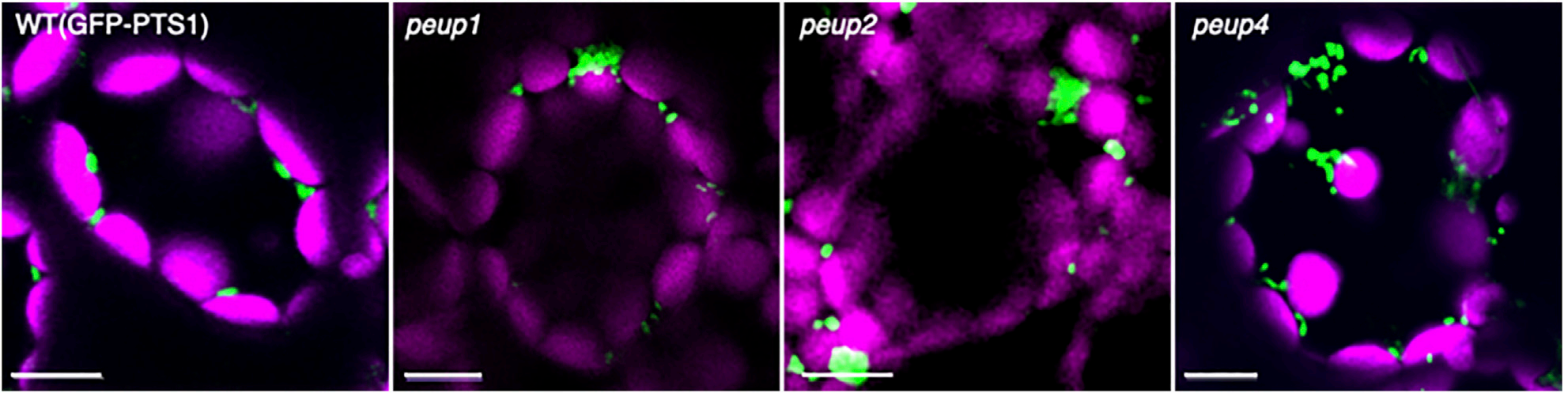
Peroxisome aggregation in *peup* mutants. Representative images of peroxisomes (green) and chloroplasts (magenta) in leaf mesophyll cells of the WT plant and *peup* mutants (Shibata et al., 2013; Goto-Yamada et al., 2019). Peroxisomes associate with chloroplasts in the WT plant, whereas peroxisomes partially form aggregates in *peup* mutants. Bars, 10 µm.

It is crucial to study mutants in order to understand the biological significance of peroxisome movement and positioning (interactions with other organelles) for cellular function. *A. thaliana peup* mutants were isolated from the EMS-mutagenized seed pools that were used to obtain *apem* mutants by the following method (Table 1; Shibata et al., 2013; Goto-Yamada et al., 2019). Leaves of the mutant lines were put on an agar plate under 100 μmol m^−2^ s^−1^ light to distribute chloroplasts perpendicular in leaf mesophyll cells (Kagawa et al., 2001; Oikawa et al., 2008; Wada and Kong, 2018). In WT cells, peroxisomes reside in a similar location as chloroplasts at the cell periphery because they mostly interact with chloroplasts (Oikawa et al., 2015). It was expected that if the mutants were defective in the peroxisome motility system that regulates peroxisome localization or in tethering factors that connect a peroxisome with a chloroplast, peroxisomes would exhibit abnormal positioning or remain in the cytosol distant from the chloroplast. About 10,000 plants were screened under a fluorescence microscope, and more than 50 *peup* mutants, which displayed peroxisome aggregation and diffuse localization in the cytosol due to a defect in interactions with chloroplasts, were obtained. Of them, *peup1*, *peup2*, and *peup4* exhibited remarkable peroxisome aggregation and an increased number of peroxisomes (Figure 3; Shibata et al., 2013). Furthermore, the mutants displayed earlier senescence than WT plants in normal air conditions (Shibata et al., 2013; Yoshimoto et al., 2014). *PEUP1*, *PEUP2*, and *PUEP4* encode autophagy-related (ATG) 2, ATG18a, and ATG7 proteins, respectively (Table 1; Shibata et al., 2013). These mutants accumulated undegraded peroxisomes containing inactive catalase aggregates, which were observed as high-density regions in peroxisomes in electron micrographs (Shibata et al., 2013). The undegraded peroxisomes are defective in interactions with chloroplasts and movement in the cytosol (Yoshimoto et al., 2014).

Other *PEUP* genes, *PEUP17* and *PEUP22*, were recently revealed to encode ATG5 and ATG7, respectively (Table 1). Analysis of *peup17* and *peup22* demonstrated that sucrose starvation induces a type of microautophagy in root tip cells and that *ATG* genes are involved in this process (Goto-Yamada et al., 2019). Peroxisomes in other *peup* mutants are spherical with reduced motility or form small aggregates with aberrant motility. These mutants are expected to have defects in gene products that regulate the interaction between a peroxisome and a chloroplast or peroxisome mobility, such as tethering factors or receptors of motor proteins. They will be useful materials to study peroxisome quality control via autophagy and organelle interactions.

### 2.4 Conclusion of Analyses of *apem* and *peup* Mutants

**FIGURE 4:**
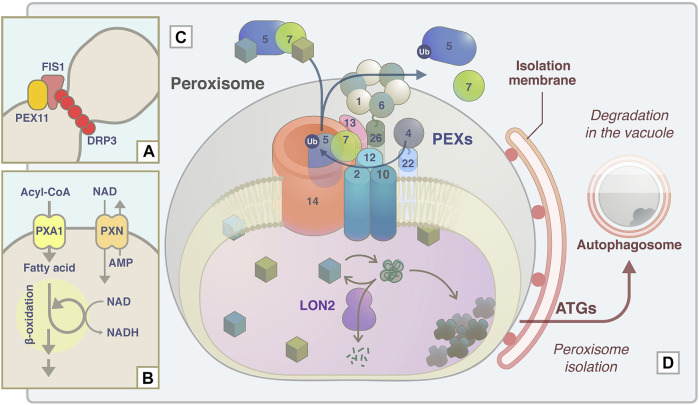
Schematic model of APEM protein functions in peroxisome proliferation, lipid metabolism, protein transport machinery, and quality control. **(A)** During peroxisome fission, DRP3A/APEM1 is recruited to the peroxisome division site together with DRP3B in a PEX11- and FIS1-dependent manner (Kao et al., 2018). DRP proteins are polymerized and constrict to divide peroxisomes. **(B)** PXN/APEM3 import NAD into the peroxisomal matrix and this is required for optimal fatty acid β-oxidation. **(C)** Peroxisomal matrix proteins are captured by the receptor PEX5 or PEX7. The PEX5-PEX7-cargo complex translocates to peroxisomes by binding to the docking complex consisting of PEX14 and PEX13/APEM2. The E2 ubiquitin ligase PEX4 and the E3 ligase PEX2/PEX10/PEX12 supposedly ubiquitinate PEX5 to export it from the peroxisomal membrane with/without the force generated by the APEM9/PEX15/PEX26-tethered AAA-ATPase PEX1-PEX6 complex. Experimental data support the interactions between PEX13 and PEX7 (Mano et al., 2006), PEX13 and PEX15/PEX26 (Li et al., 2014), and PEX7 and PEX12 (Singh et al., 2009). **(D)** Damaged and/or unwanted peroxisomal proteins are supposedly maintained or degraded by the chaperone/protease activity of LON2/APEM10 protein. Excess damaged proteins accumulate inside peroxisomes. Peroxisomes become oxidative upon catalase inactivation and aggregation, and these peroxisomes are targeted for pexophagy to be degraded in the vacuole (Shibata et al., 2013). ATG proteins, including ATG2/PEUP1, ATG18A/PEUP2, ATG7/PEUP4/PEUP22, and ATG5/PEUP17, are involved in this process.

The *apem* and *peup* mutant series were isolated based on the imaging technique in our laboratory and are summarized in Figure 4. APEM1/DRP3A was identified as a major component of the peroxisome division machinery. APEM2/PEX13, APEM4/PEX12, and APEM9/PEX15/PEX26 were identified as a group of peroxisome biogenesis factors. Like in animals, many plant PEX mutants with T-DNA insertions causing complete protein dysfunction display lethality, as reported in studies of PEX2, PEX10, PEX12, PEX13, APEM9/PEX15/PEX26, and PEX16 (Lin et al., 1999; Hu et al., 2002; Sparkes et al., 2003; Fan et al., 2005; Boisson-Dernier et al., 2008; Goto et al., 2011). Therefore, partial loss of function of each PEX, rather than complete abolition of PEX function, is desirable to study plant peroxisomes. EMS-induced mutagenesis causes single nucleotide substitutions and is therefore expected to induce a milder loss of function than null mutations. The functions of the regions of PEX13 and PEX12 that are affected by the *apem2* and *apem4* mutations, respectively, are unknown, and further analysis is required to understand how these regions contribute to the functions of the proteins and their interactions with other proteins. APME9 is functionally equivalent to PEX15/PEX26 found in fungi and mammals. It has no detectable sequence similarity to PEX15 or PEX26, which emphasizes the major advantage of the forward genetic approach, i.e., the discovery of novel factors. Analysis of APEM10/LON2 revealed that its chaperone and protease functions, as well as autophagy acting in concert with these functions, are required for peroxisome quality control. APEM3/PMP38/PXN is a membrane transporter that supplies NAD^+^ to the peroxisomal matrix. Depletion of NAD^+^ induces accumulation of fatty acids, and their toxicity may result in enlargement of peroxisomes in *apem3*. The study of PEUP1/ATG2, PEUP2/ATG18A, and PEUP4/ATG7 provided evidence that damaged peroxisomes accumulate a massive amount of inactivated catalase, and abnormal oxidative conditions induce pexophagy. In addition, the study of PEUP17/ATG5 and PEUP22/ATG7 has shed light on a new type of microautophagy induced by starvation.

### 2.5 Other Imaging Analyses of Peroxisomes

#### 2.5.1 Imaging-Based Mutant Screening Other Than That of *apem* and *peup* Mutants

The screening strategy, e.g., the parental strain to be mutagenized and the criteria for isolation of mutants, depends on the experiments. The most reported imaging-based approach is mutagenesis and screening of strains with visualized peroxisomes as described in the above section. Zhang and Hu. (2009) reported the screening and identification of *A. thaliana peroxisome division/proliferation deficient* (*pdd*) mutants to identify factors involved in peroxisome division and proliferation pathways. *pdd* mutants were isolated from parental EMS-mutagenized *A. thaliana*, which expressed 35S promoter-driven YFP-PTS1. They identified *pdd1* and *pdd2* as DRP3A alleles (Aung and Hu, 2009; Zhang and Hu, 2009). Rinaldi et al. (2016) reported a massive number of mutants, with 34 novel alleles of 15 genes involved in oil body mobilization, fatty acid β-oxidation, the glyoxylate cycle, peroxisome fission, and pexophagy (Rinaldi et al., 2016). These mutants were isolated from EMS-mutagenized GFP-PTS1-expressing plants, and the mutations were determined by a combination of map-based cloning and direct or whole genome sequencing. Although it had been reported that deficiencies in enzymes involved in peroxisomal β-oxidation led to swollen peroxisomes, this had not been proven. One of the main achievements of this report is genetically proving that accumulation of fatty acids inside peroxisomes leads to enlargement of peroxisomes using a number of isolated mutants defective in peroxisomal metabolism and transporters (Rinaldi et al., 2016). The visualization of peroxisomes is also effective in mammalian cells to isolate peroxisome-deficient mutants. Ghaedi et al. (1999) generated Chinese Hamster Ovary cells stably transformed with GFP-PTS1 or PTS2-GFP, and mutagenized these cells with N-methyl-N’-nitro-N-nitrosoguanidine. The mutant phenotypes were investigated by observation under a fluorescence microscope (Ghaedi et al., 1999). Another group employed a similar technique, and these studies identified several genes related to peroxisome biogenesis (Ito et al., 2000; Ghaedi and Fujiki, 2008). Comprehensive, imaging-based, large-scale screening has been achieved in yeast. Yeast is a very useful tool for functional analysis of proteins because of the ease of genetic analyses and the large number of established analytical tools. A collection of gene deletions covering 96% of yeast open reading frames, called a modified synthetic genetic array (SGA), is available, and automated screening is also possible (Giaever et al., 2002). Wolinski et al. (2009) established an experimental platform that can be connected to a SGA, enabling qualitative, quantitative, and automated large-scale analysis of GFP-labeled peroxisomes in yeast cells (Wolinski et al., 2009). The technique identified two novel genes that had not been previously linked to peroxisome biogenesis as well as all known factors required for PTS1-dependent protein transport. Cohen et al. (2014) employed dual reporters to visualize peroxisomes. Cherry fused with PTS1 (Cherry-PTS1) and GFP-tagged peroxisomal membrane protein Ant1 (GFP-Ant1) allow assessment of the efficiency of not only matrix protein sorting but also membrane protein transport and peroxisome formation (Cohen et al., 2014). Use of a combination of the SGA approach and multiple reporters identified a P-type ATPase and ion transporter in the ER membrane (Spf1), which is required for delivery of peroxisomal membrane proteins to peroxisomes, and revealed that peroxisomes localize in close contact with mitochondria and ER (Cohen et al., 2014).

In addition to mutant screening using strains with visualized peroxisomes, mutant screenings focusing on specific peroxisomal factors or phenomena have also been reported. Burkhart et al. (2013) focused on degradation of peroxisomal enzymes in glyoxysomes, which are a type of peroxisomes in which the glyoxylate cycle occurs and are found in cotyledons during early germination of seedlings (Burkhart et al., 2013). ISOCITRATE LYASE (ICL), a glyoxysomal enzyme, is required for lipid conversion to sucrose during post-germinative growth and becomes unnecessary once photosynthesis starts in seedlings (Nishimura et al., 1982; Titus and Becker, 1985). To identify components required for degradation of unwanted peroxisomal matrix proteins, a transgenic plant expressing *GFP-ICL* was mutagenized with EMS. Mutants that retained GFP-ICL longer than the WT, named *persistent GFP-ICL fluorescence* (*pfl*) mutants, were isolated (Burkhart et al., 2013). In the screening, proteins involved in the docking complex (PEX14) and recycling complex (PEX2, PEX6, and PEX10) of the matrix protein transport machinery and a β-oxidation enzyme (PED1/KAT2) were identified from the *pfl* mutants (Burkhart et al., 2013; Burkhart et al., 2014). From these analyses, the authors concluded that efficient degradation of peroxisomal matrix proteins requires proteins to be sorted inside peroxisomes and also seems to require an appropriate metabolic level of peroxisomes and the entire peroxisomal protein transport system (Burkhart et al., 2013). A unique and large-scale screening was performed in yeast to study the priority of peroxisomal protein targeting. Peroxisomal proteins containing the PTS1 targeting signal are captured by the receptor PEX5 and transported to peroxisomes. If the level of cargo becomes high, the occupancy of PEX5 increases and only proteins with a high targeting priority will localize to peroxisomes. Rosenthal et al. (2020) generated yeast strains that express varying levels of PTS1 fused to mCherry (mCherry-SKL) by changing the copy number of mCherry-SKL in the construct (Rosenthal et al., 2020). Around 90 strains expressing peroxisomal proteins tagged with GFP were transformed with low or high levels of mCherry-SKL, and the localization of GFP to peroxisomes was measured in each strain using an automated microscopy platform.

#### 2.5.2 Visualization of Peroxisomes Using Other Imaging Technical Methods

In the correlative light and electron microscopy (CLEM) method, fluorescence and dyes in a sample are observed with an optical microscope, and then the same area is observed with an electron microscope (Razi and Tooze, 2009; Jahn et al., 2012). Although various CLEM methods have been developed and reported, they have mainly used cultured animal cells, and there are few reports on methods suitable for plant tissues and cells. Toyooka (2016) developed a new CLEM method to accurately capture the localization of fluorescently labeled biomolecules in plant tissues and cells at high resolution, and applied the method to *A. thaliana* with GFP-labeled peroxisomes (Toyooka, 2016). In yeast *Hansenula polymorpha*, the peroxisome-vacuole contact site was visualized using the CLEM method, and Pex3 is shown to be involved in the formation of peroxisome-vacuole contact sites (Wu et al., 2019). Bykov et al. (2019) developed a new methodology, MultiCLEM, to allow systematic, parallel, high-throughput screening for traits using the CLEM with computer image analysis (Bykov et al., 2019). By applying MultiCLEM to different yeast strains with GFP-labelled peroxisomes, they successfully identified peroxisomes in both fluorescence and electron microscopic images (Bykov et al., 2019). Since this methodology apparently can be scaled up to higher throughputs, not limited to yeast, it is expected to enable electron microscopy a powerful screening method.

Three-dimensional ultrastructural images with quantitative information can be reconstructed from image data obtained by transmission electron microscopy or focused ion beam scanning electron microscopy (FIB-SEM). Recently, Zechmann et al. (2021) reported that quantitative changes of the volumes of viral inclusion bodies, chloroplast fine structures, mitochondria, and peroxisomes using reconstituted 3D image data (Zechmann et al., 2021). They reconstituted 3D images during the process of *Tobacco mosaic virus* and *Zucchini yellow mosaic virus* infection in tobacco and pumpkin plants from serial sections obtained by transmission electron microscopy and extracted quantitative information on the size and number of peroxisomes and other organelles (Zechmann et al., 2021). In mouse liver hepatocytes, the wrappER, a curved wrapping type of rough ER accumulates fatty acid and fatty acid-binding proteins of the lipocalin family and regulates intracellular and systemic lipid flux by establishing extensive contact with almost all mitochondria. Ilacqua et al. (2022) showed that the wrappER contacts with peroxisomes in addition to mitochondria by analyzing a large portion of the cell volume of the hepatocytes by serial section electron tomography coupled to 3D reconstruction. Xu et al. (2017) reported an extended FIB-SEM system for high volume 3D imaging suitable for connectomics (Xu et al., 2017). Using this new system, the authors have successfully imaged large, complex samples of mammalian neural tissue, *Drosophila* brain, and *Chlamydomonas reinhardtii* in entirety with sufficient detail to allow high-quality reconstruction of connections. The introduction of these new imaging techniques is expected to make it possible to analyze peroxisome dynamics at higher resolution, more easily, and with a larger volume of data.

## 3 Evolution of Peroxisome Dynamics in Land Plants

### 3.1 The Liverwort *M. polymorpha* as a Model

**FIGURE 5:**
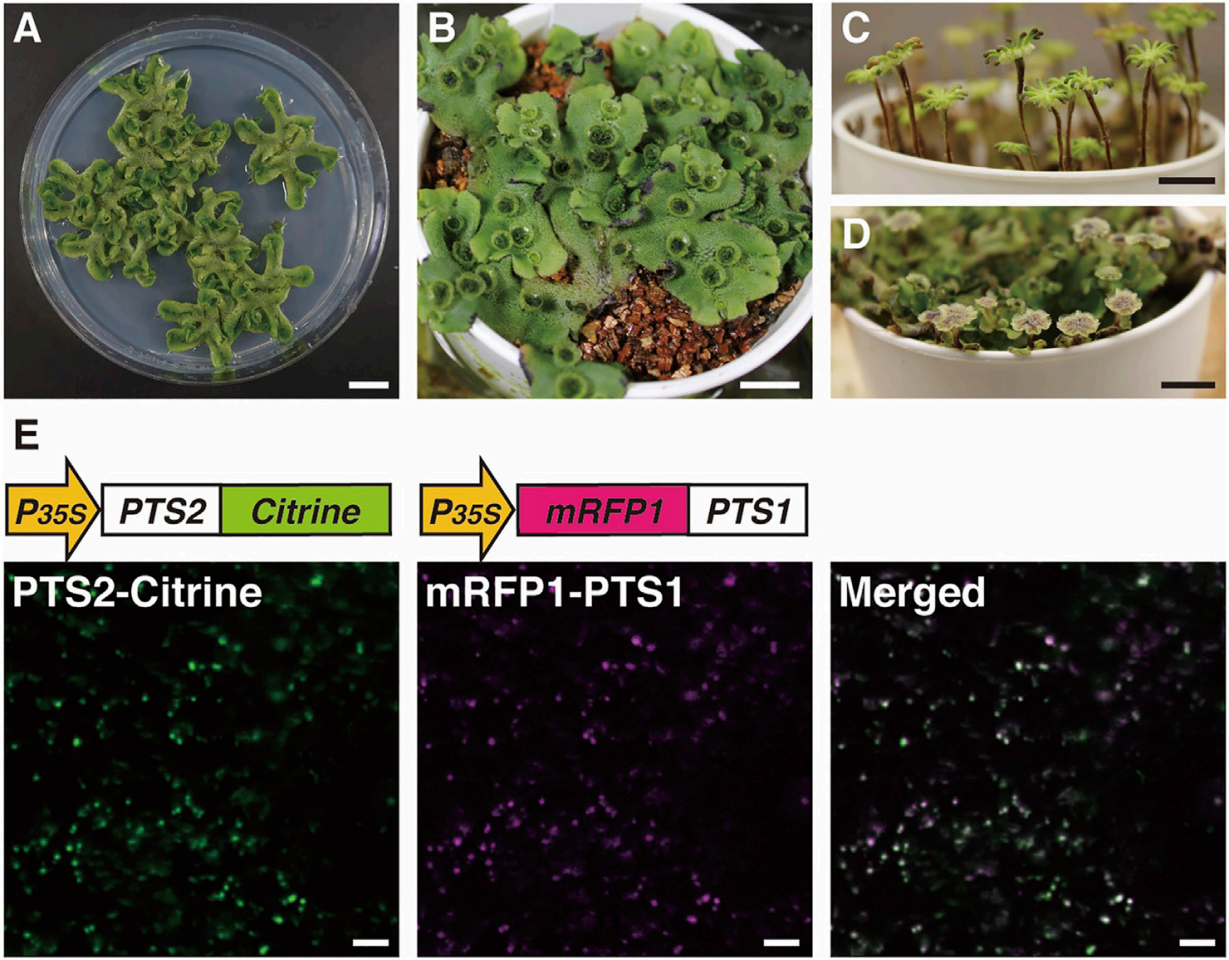
Images of *M. polymorpha* and visualization of peroxisomes using Citrine- and mRFP1-fused proteins. Vegetative haploid life form (thallus) on agar plate **(A)** and vermiculite **(B)** (Shimamura, 2016). Female **(C)** and male **(D)** sexual organs from the haploid thallus of a female plant or a male plant, respectively (Chiyoda et al., 2008; Inoue et al., 2019). Bars, 1 cm. **(E)** Fluorescence in peroxisomes was observed in thallus epidermal cells expressing both *pro35S:PTS2-Citrine* and *pro35S:mRFP1-PTS1* genes (Mano et al., 2018). Bars, 10 μm.

Our current understanding of the biogenesis and function of peroxisomes in land plants is largely based on the studies using *A. thaliana* as described above. To obtain more insights into the evolution of peroxisome dynamics in land plants, yet another model plant that is divergent from *A. thaliana* is needed: the liverwort *M. polymorpha*. This bryophyte species is an early diverging land plant and thus retains features of ancestral land plants. Its main form during its gametophyte-dominant life cycle is a complex thalloid structure with cupules containing gemmae for asexual propagation and rhizoids on the ventral and dorsal surfaces, respectively (Figures 5A,B; Shimamura, 2016). Like many other bryophyte species, *M. polymorpha* is dioicous and has heteromorphic sex chromosomes: U with the sex determining gene for female and V for male (Haupt, 1932; Bowman et al., 2017; Montgomery et al., 2020; Iwasaki et al., 2021). Under long-day conditions enriched with far-red light, *M. polymorpha* initiates transition from the vegetative to reproductive phase, generating sexual organs (Figures 5C,D; Chiyoda et al., 2008; Inoue et al., 2019). Motile sperm are released from a male reproductive organ, the antheridiophore, and navigate to a female reproductive organ, the archegoniophore. Sperm can be readily collected from male plants and applied to female plants, meaning genetic crosses of *M. polymorpha* are easily performed. After fertilization, a zygote continues mitotic division to form a diploid multicellular sporangium. Meiotic division of spore mother cells in each sporangium produces as many as 300,000 haploid spores (O'Hanlon, 1926), which is advantageous for forward genetics by mutagenesis. The genome of *M. polymorpha* has a set of regulatory systems comparable with that in angiosperms but in a remarkably less redundant form, presumably representing the situation in ancestral land plants (Bowman et al., 2017). Its low genetic redundancy, together with the molecular and genetic tools described below, makes *M. polymorpha* a model plant of choice for both forward and reverse genetics to elucidate the molecular machineries that operate in land plants (Ishizaki et al., 2016; Sauret-Güeto et al., 2020; Kohchi et al., 2021).

Genetic transformation of *M. polymorpha* has been well-established (Chiyoda et al., 2008; Ishizaki et al., 2008; Kubota et al., 2013) and continuously improved (Tsuboyama-Tanaka and Kodama, 2015; Tsuboyama and Kodama, 2018; Iwakawa et al., 2021; Seo et al., 2021). A wide range of gateway vectors for *Agrobacterium*-mediated transformation have been developed and made available for simple gene transfer, conditional gene expression and deletion (Nishihama et al., 2016), reporter assays (Ishizaki et al., 2015; Mano et al., 2018), and CRISPR/Cas9-mediated genome editing (Sugano et al., 2018; Sugano and Nishihama, 2018). Gene targeting including knock-in by homologous recombination is also feasible (Ishizaki et al., 2013; Yamaoka et al., 2018; Yasui et al., 2019; Kato et al., 2020).

There are web-based databases where genomic and related resources can be accessed, including MarpolBase (https://marchantia.info, Bowman et al., 2017; Montgomery et al., 2020), Phytozome (https://phytozome-next.jgi.doe.gov/info/Mpolymorpha_v3_1), and Ensembl Plants (https://plants.ensembl.org/Marchantia_polymorpha/Info/Index). MarpolBase is an up-to-date comprehensive site dedicated to *M. polymorpha* studies, where researchers can browse, search, and retrieve sequences and genes, design CRISPR/Cas9 target sites, and browse lists of *M. polymorpha*-related literature. The guideline for *M. polymorpha* gene nomenclature is also available at this site (Bowman, 2016).

### 3.2 Visualization of Peroxisomes in *M. polymorpha*

As described in Section 2, visualization of peroxisomes with fluorescent proteins in *A. thaliana* greatly helped to unveil the molecular dynamics of plant peroxisomes. To investigate whether the regulatory mechanisms of peroxisome dynamics that were clarified using *A. thaliana* are conserved among plant species or species-specific, transgenic *M. polymorpha* plants expressing *Citrine-PTS1*, *mRFP1-PTS1*, and *PTS2-Citrine* have been generated (Figure 5E; Ogasawara et al., 2013; Kimura and Kodama, 2016; Mano et al., 2018). Research using these transgenic plants revealed that the morphology, size, and movement of peroxisomes in *M. polymorpha* resemble those in *A. thaliana* (Mano et al., 2018). Moreover, peroxisomes relocated from the periclinal cell wall to the anticlinal cell wall after cold treatment (Ogasawara et al., 2013) and this relocation was mediated via actin filaments (Kimura and Kodama, 2016), suggesting that similar mechanisms mediate subcellular positioning of peroxisomes in response to environmental stimuli in *M. polymorpha* and *A. thaliana* (Oikawa et al., 2015). To generate transgenic *M. polymorpha* with visualized peroxisomes, PTS1 or PTS2 was fused to fluorescent proteins, meaning that both PTS1- and PTS2-dependent protein transport pathways could be analyzed. Genes encoding proteins with high similarities to PEX5 and PEX7, which are receptors for PTS1 and PTS2, respectively, are present in the *M. polymorpha* genome (Table 2). This indicates that both pathways were required from the beginning of evolution of land plants.

**TABLE 2:** *PEX* genes in representative land plants and algae.

Function	Name	*Arabidopsis thaliana* (Dicot)	*Marchantia polymorpha* (Liverwort)	*Mesotaenium endlicherianum* (Zygnematales)	*Klebsormidium nitens* (Klebsormidiales)
AAA-ATPase	PEX1	At5g08470	Mp6g06650.1	ME000591S08541	kfl00001_0640
RING finger protein	PEX2	At1g79810	Mp6g00800.1	ME000422S07096	kfl00019_0620
Membrane protein import	PEX3	At3g18160	Mp7g11800.1	ME000132S00389	kfl00083_0070
		At1g48635			
Ubiquitin-conjugating enzyme	PEX4	At5g25760	Mp1g00960.1	ME000123S00236	kfl00180_0090
Receptor for PTS1 proteins	PEX5	At5g56290	Mp8g01780.1	ME000013S00808	kfl00041_0250
AAA-ATPase	PEX6	At1g03000	Mp3g11610.1	ME000232S03803	kfl00209_0140
Receptor for PTS2 proteins	PEX7	At1g29260	Mp8g16810.1	ME000671S09013	kfl00007_0620
RING finger protein	PEX10	At2g26350	Mp1g01820.1	^a^ME000464S07766 ^a^ME000464S07763	kfl00169_0180
Peroxisome division/proliferation	PEX11a	At1g47750	Mp1g28560.1	ME000109S10892	kfl00012_0580
	PEX11b	At3g47430	Mp1g26710.1	ME000184S02250	—
	PEX11c	At1g01820	Mp8g02510.1	ME000659S08975	kfl00038_0110
	PEX11d	At2g45740		
	PEX11e	At3g61070		
RING finger protein	PEX12	At3g04460	Mp6g05650.1	—	kfl00469_0030
Receptor docking	PEX13	At3g07560	Mp4g02320.1	—	kfl00041_0020
Receptor docking	PEX14	At5g62810	Mp7g18230.1	ME000301S05072	kfl00067_0040
Membrane protein import	PEX16	At2g45690	Mp6g13850.1	ME000020S03138	kfl00150_0180
Membrane protein import	PEX19	At3g03490 At5g17550	Mp6g19710.1	ME000172S01963	kfl00057_0390
Membrane anchor of PEX4	PEX22	At3g21865	Mp3g11230.1	ME000134S00709	kfl00100_0220
Membrane anchor for PEX1-PEX6 complex	APEM9	At3g10572	Mp2g15620.1	—	kfl00146_0030

a Likely divided by a sequencing gap, the central region of the intact gene is likely located within the gap between the apparent two gene models. Modified from Table 1 in Cross et al. (2016).

### 3.3 Bioinformatics Analysis to Identify Peroxisomal Genes

**FIGURE 6:**
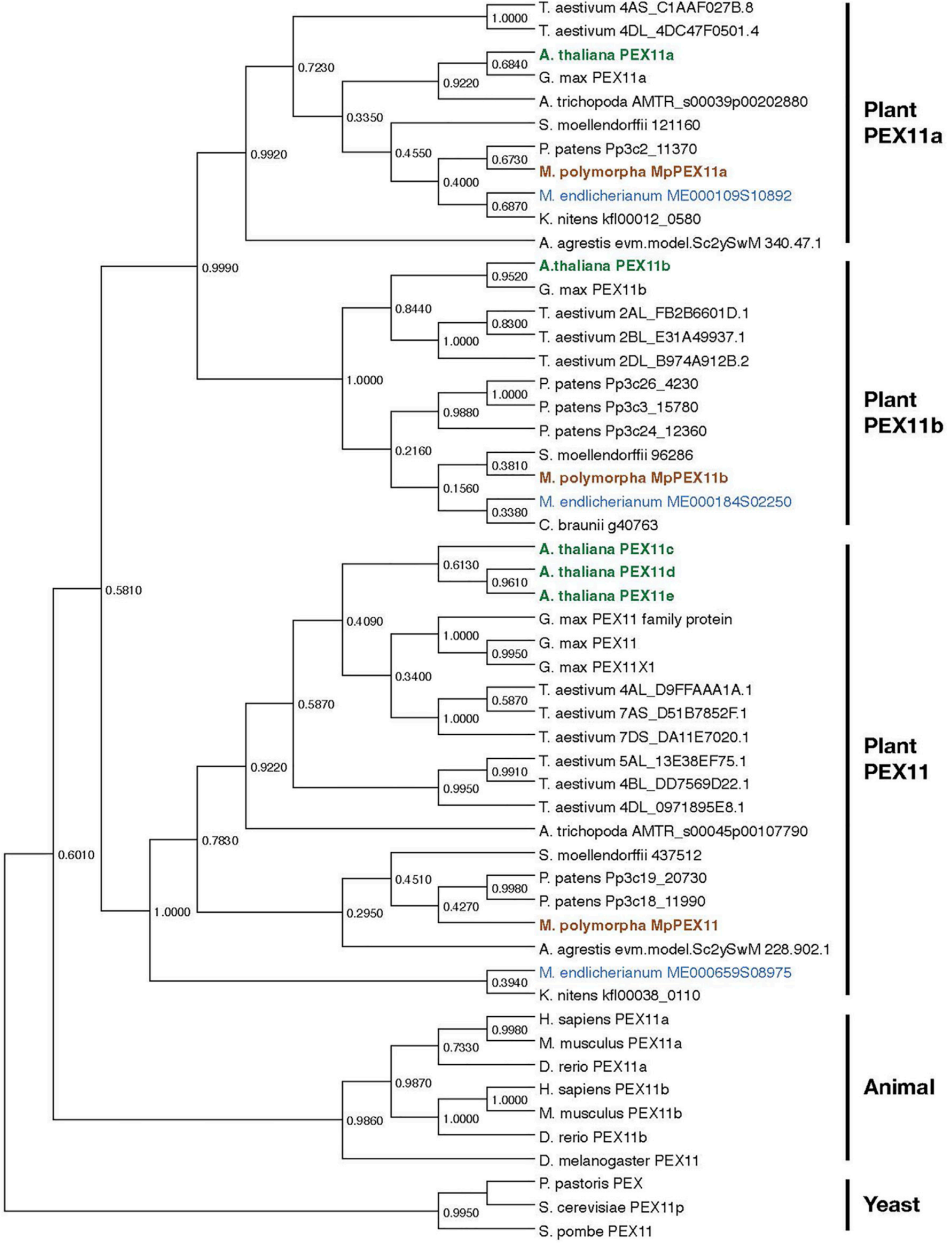
Phylogenetic relationships of PEX11 subfamilies. The numbers are the proportion of trees in which the associated sequences cluster together. Sequences of *A. thaliana*, *M. polymorpha*, and *M. endlicherianum* are colored as representatives from among angiosperms, bryophytes, and algae, respectively. The phylogenetic tree for PEX11 homologs was inferred using the Maximum Likelihood method and JTT matrix-based model (Jones et al., 1992) with MEGA11 (Stecher et al., 2020; Tamura et al., 2021). All positions with less than 95% site coverage were eliminated, i.e., fewer than 5% alignment gaps, missing data, and ambiguous bases were allowed at any position (partial deletion option). Orthologue sequences in plants were obtained from the datasets for *C. braunii* (Nishiyama et al., 2018), *M. endlicherianum* (Cheng et al., 2019), *K. nitens* (Hori et al., 2014), *M. polymorpha* (Montgomery et al., 2020), *P. patens* (Lang et al., 2018), *A. agrestis* (Li et al., 2020), *S. moellendorffii* (Banks et al., 2011), *A. trichopoda* (Amborella Genome Project, 2013), *A. thaliana* (Berardini et al., 2015), *G. max* (Schmutz et al., 2010), and *T. aestivum* (International Wheat Genome Sequencing Consortium, 2014). Other PEX11 species used in this analysis are *P. pastoris* (ANZ76138.1), S. *cerevisiae* (AJT75217.1), *S. pombe* (NP_595177.1), *D. melanogaster* (NP_611071.1), *D. rerio* (NP_001096590.1 and NP_001039319.1), *H. sapiens* (NP_003838.1 and NP_003837.1), and *M. musculus* (NP_035198.1 and NP_001155859.1).

Peroxisome biogenesis requires a set of specialized proteins, peroxins, encoded by *PEX* genes. *A. thaliana* has 22 *PEX* genes (Table 2; Cross et al., 2016), while *M. polymorpha* has 18. Most *PEX* genes, except for *PEX3*, *PEX11c*/*d*/*e*, and *PEX19*, in *A. thaliana* have a single counterpart in *M. polymorpha*. Duplication and triplication of *PEX3*/*PEX19* and *PEX11*, respectively, in *A. thaliana* explains why there are more *PEX* genes than in *M. polymorpha*. Phylogenetic analysis revealed that duplication of *PEX11a* and *PEX11b* predates the divergence of Zygnematales and Embryophytes, and the divergence of *PEX11a*/*b* and *PEX11c*/*d*/*e* likely occurred even earlier (Figure 6), suggesting that *PEX11* should be further categorized into three subclasses, *PEX11a*, *PEX11b*, and others, increasing the total number of PEX subclasses in land plants to 18. It should be noted that *M. polymorpha* has the complete set of 18 *PEX* genes without duplication, which makes it suitable for functional and evolutionary analyses. The set of *PEX* genes in *M. polymorpha* appears to have been already established in the common ancestor of Zygnematales and Embryophytes, although there are a few missing genes in *Mesotaenium endlicherianum* and *Klebsormidium nitens*, which could be explained by secondary loss in these lineages and/or the presence of sequence gaps (Table 2).

### 3.4 Genome Editing to Analyze Peroxisome Dynamics in *M. polymorpha*

Genome editing is a powerful tool for functional analysis of gene products and is applied in various organisms. CRISPR/Cas9-based vectors with high efficiency have been established and used in *M. polymorpha* (Sugano et al., 2018; Sugano and Nishihama, 2018). As described above, bioinformatics analysis of peroxisomal genes in *A. thaliana* revealed the presence of orthologous genes in the *M. polymorpha* genome. For example, *Mp6g18570* shows high similarity to *At3g19190*, which is the responsible gene in the *peup1/atg2* mutant (Shibata et al., 2013). Norizuki et al. (2019) performed CRISPR/Cas9-based genome editing of several *M. polymorpha ATG* genes including *Mp6g18570* (Norizuki et al., 2019). *Mp6g18570*-edited *M. polymorpha* exhibited earlier senescence than the WT plant (Norizuki et al., 2019), consistent with the phenotype of the *A. thaliana peup1/atg2* mutant. PEUP1/ATG2 has a role in autophagy (Shibata et al., 2013), demonstrating the existence of a similar degradation system in *M. polymorpha*. As described above, some *A. thaliana* peroxisomal genes, such as *PEX3*, *PEX11*, and *PEX19*, constitute a gene family, and they have single counterparts in *M. polymorpha*. This is true of other genes encoding metabolic enzymes that function inside peroxisomes; the number of genes constituting the family is decreased in *M. polymorpha*. Therefore, *M. polymorpha* is a good material to investigate the functions of gene products in peroxisome research because the generation of mutants with knockout and/or knockdown of gene products requires manipulation of fewer genes and thus is easier. This approach will uncover the mechanisms underlying peroxisome dynamics and diversification of peroxisomes during the evolution of plants, accelerating peroxisome research.

## 4 Conclusion

*A. thaliana* mutants with peroxisomes defects that were obtained using transgenic *A. thaliana* with visualized peroxisomes as a parent material have greatly helped to identify essential components for regulation of peroxisome dynamics. A similar approach in which plants with visualized peroxisomes, including *M. polymorpha*, as a parent material are randomly mutagenized can be used to obtain valuable mutants based on imaging analysis. Together with plants with visualized peroxisomes, genome editing of *M. polymorpha* target genes identified by bioinformatic analysis can be performed to investigate the dynamics and diversity of peroxisomes in land plants.

Screening and phenotyping of a large number of mutants take an enormous amount of time. Automated screening, as in the yeast example described above, could dramatically reduce the experimental time. Li et al. (2021) recently established Deep Learning of the Morphology of Organelles (DeepLearnMOR), which can categorize mutant phenotypes and identify an abnormal morphology with 97% accuracy (Li et al., 2021), and can be used to accurately and quantitatively analyze phenotypes. It is hoped that this new technology will accelerate and advance identification of mutants in peroxisome biology in the near future.

## 5 Future Prospects for Plant Peroxisome Research

As described in this review, imaging-based mutant screening has identified various factors involved in peroxisome dynamics and have elucidated their molecular mechanisms. However, there are still unresolved issues in plant peroxisome research that remain to be addressed. For example, autophagosomes have been reported to access damaged peroxisomes, but how autophagosomes detect internal peroxisomal abnormalities and recognize only abnormal peroxisomes in plants is not understood. Peroxisomes interact with other organelles, such as endoplasmic reticulum, chloroplasts, and mitochondria, at membrane contact sites (MCS) between organelles to exchange metabolites and signals, thereby playing a role in maintaining cellular homeostasis (Prinz, 2014; Perico and Sparkes, 2018). However, the mechanism of MCS formation between peroxisomes and other organelles is not yet fully understood. The morphology and movement of plant peroxisomes are influenced by ROS induced by environmental stresses such as high light and high temperature. ROS alter the organelle membrane structure, resulting in the formation of peroxules from peroxisomes (Mathur, 2021). However, little is known about the dynamics of peroxisomal membrane lipids. Recent studies have also revealed that peroxisomes have essential roles in reproductive processes, such as pollen fertility, male-female recognition, and embryo development after fertilization (Sparkes et al., 2003; Fan et al., 2005; Boisson-Dernier et al., 2008; Goto et al., 2011; Goto-Yamada et al., 2014b). However, the roles of peroxisomes and molecular mechanisms underlying their functions in the reproductive process are less well understood than in the analysis of roots and leaves.

To address the above issues, it is necessary to introduce new imaging techniques such as super-resolution microscopy analysis using the stimulated release depletion method and structured illumination microscopy combined with chemical approaches (Ovečka et al., 2022). In addition, quantitative methods using fluorescent probes to visualize ROS (de Torres Zabala et al., 2015), redox state (Exposito-Rodriguez et al., 2017), ATP (Voon et al., 2018), and NADH/NAD^+^ (Lim et al., 2020), split fluorescent proteins, and FRET techniques (Shai et al., 2018; Vallese et al., 2020) are also expected to be available for isolation of interesting peroxisome mutants. Furthermore, combining femtosecond laser and optical tweezers (Oikawa et al., 2015; Gao et al., 2016) with biochemical approaches such as proteome (or lipidome) analyses by immunoprecipitation and mass spectrometry is expected to be useful in the search for proteins that mediate peroxisome tethering with other cellular structures. Further advances in imaging technology are expected to elucidate various peroxisome-mediated biological phenomena and the molecular mechanisms that control them, such as the interaction between peroxisomes and other organelles.

## Supplementary Material

Supplementary Movie S1 Movement of peroxisomes in root cells. Time-lapse images were collected every 2 s for 1 min. Most peroxisomes show non-directional and random motion. The movie is shown at double speed.
